# Proteome-Wide Fragment-Based Ligand and Target Discovery

**DOI:** 10.1002/ijch.202200098

**Published:** 2023-02-08

**Authors:** Ines Forrest, Christopher G. Parker

**Affiliations:** a Department of Chemistry, The Scripps Research Institute, La Jolla, CA 92037, USA

**Keywords:** Activity-Based Protein Profiling, Chemical Proteomics, Fragment-Based Ligand Discovery, Ligandability, Photoaffinity Labeling

## Abstract

Chemical probes are invaluable tools to investigate biological processes and can serve as lead molecules for the development of new therapies. However, despite their utility, only a fraction of human proteins have selective chemical probes, and more generally, our knowledge of the “chemically-tractable” proteome is limited, leaving many potential therapeutic targets unexploited. To help address these challenges, powerful chemical proteomic approaches have recently been developed to globally survey the ability of proteins to bind small molecules (i. e., ligandability) directly in native systems. In this review, we discuss the utility of such approaches, with a focus on the integration of chemoproteomic methods with fragment-based ligand discovery (FBLD), to facilitate the broad mapping of the ligandable proteome while also providing starting points for progression into lead chemical probes.

## Introduction

Major breakthroughs in genomic sequencing and editing methods have dramatically expanded our molecular understanding of human diseases and have yielded unparalleled insight into protein function. However, in many instances, the learnings garnered from human genetics have not yet translated into new medicines. This is due to, in part, the identification of disease-related genes that code for proteins that are conventionally considered ‘tough-to-drug’, such as adaptor proteins and transcription factors. In other instances, proteins are recalcitrant to traditional small molecule screening methods or possess completely uncharacterized functions making the identification of potential chemical probes a challenge.

Conventional chemical probe discovery is typically pursued via the high-throughput screening (HTS) of large chemical libraries (~10^6^ compounds) against single protein targets (target-based), or in more complex systems, such as in whole cells or organisms (phenotype-based).^[1–2]^ Target-based screens often rely on some mechanistic insight of specific disease-related biological pathways to enable the screening of compound libraries against a single candidate target. Such screens, however, do not provide direct information about the activity of ligands in more complex biological systems (e.g., cells), where factors that regulate protein structure and function, such as subcellular localization, post-translational modifications, and protein-protein interactions can affect ligand-protein interactions. Further, such screens are restricted to a subset of proteins, owing to the fact that many targets are difficult to recombinantly express and purify, lack screenable functions, or require native environments to properly recapitulate their function and endogenous interactions.^[3–4]^ Phenotype-based screens, on the other hand, preserve native environments and are often mechanism-agnostic, lending opportunity to identify novel druggable pathways and molecules that elicit effects through non-traditional pharmacological modes of action (MoAs). However, phenotypic screening faces the challenge of identifying the molecular target(s) of active compounds, particularly in instances where hits display moderate-low potency.^[5]^ Further, phenotypic screens provide a restricted view of chemically accessible targets for any given model system. Collectively, these and related methods, have produced selective chemical probes for a modest (~1%) subset of the proteome.^[6–7]^

In recent years, powerful chemical proteomic (‘chemoproteomic’) methods have emerged that enable the discovery of ligands for proteins directly in native biological systems on a global scale. These methods typically use chemical probes that are composed of: 1) a recognition group that promotes interactions with subsets of proteins in the proteome, 2) a reactive group for covalent capture of interacting proteins, and 3) a reporter group (fluorophores, biotin, or click-chemistry enabled handles) for the detection, enrichment, and identification or visualization of interacting proteins. These features endow chemoproteomic-enabled probes with the capability to trap probe-protein interactions directly in native environments, such as living cells, preserving labile interactions that might be missed using conventional *in vitro* techniques. In addition, such probes can enrich and identify low-abundance/low-affinity interacting proteins as well as other proteins that often present technical challenges in their detection (e.g., membrane proteins). In their modern usage, these platforms use broad-profiling chemical probes to report on binding site occupancy of either covalent or non-covalent ligands across the entire proteome. To globally map ligandable proteins, recent chemoproteomic investigations have deployed small libraries (10s–100s) of fragment-based compounds. Akin to traditional fragment-based ligand discovery (FBLD) screening methods, these libraries enable the efficient exploration of chemical space while providing potential hit compounds for lead development.^[8–10]^ Here, we discuss the development of these fragment-based strategies and highlight their recent applications to expand the perceived boundaries of the ligandable proteome.

### Chemoproteomic Strategies to Broadly Survey Protein “Ligandability”

**Figure 1.**
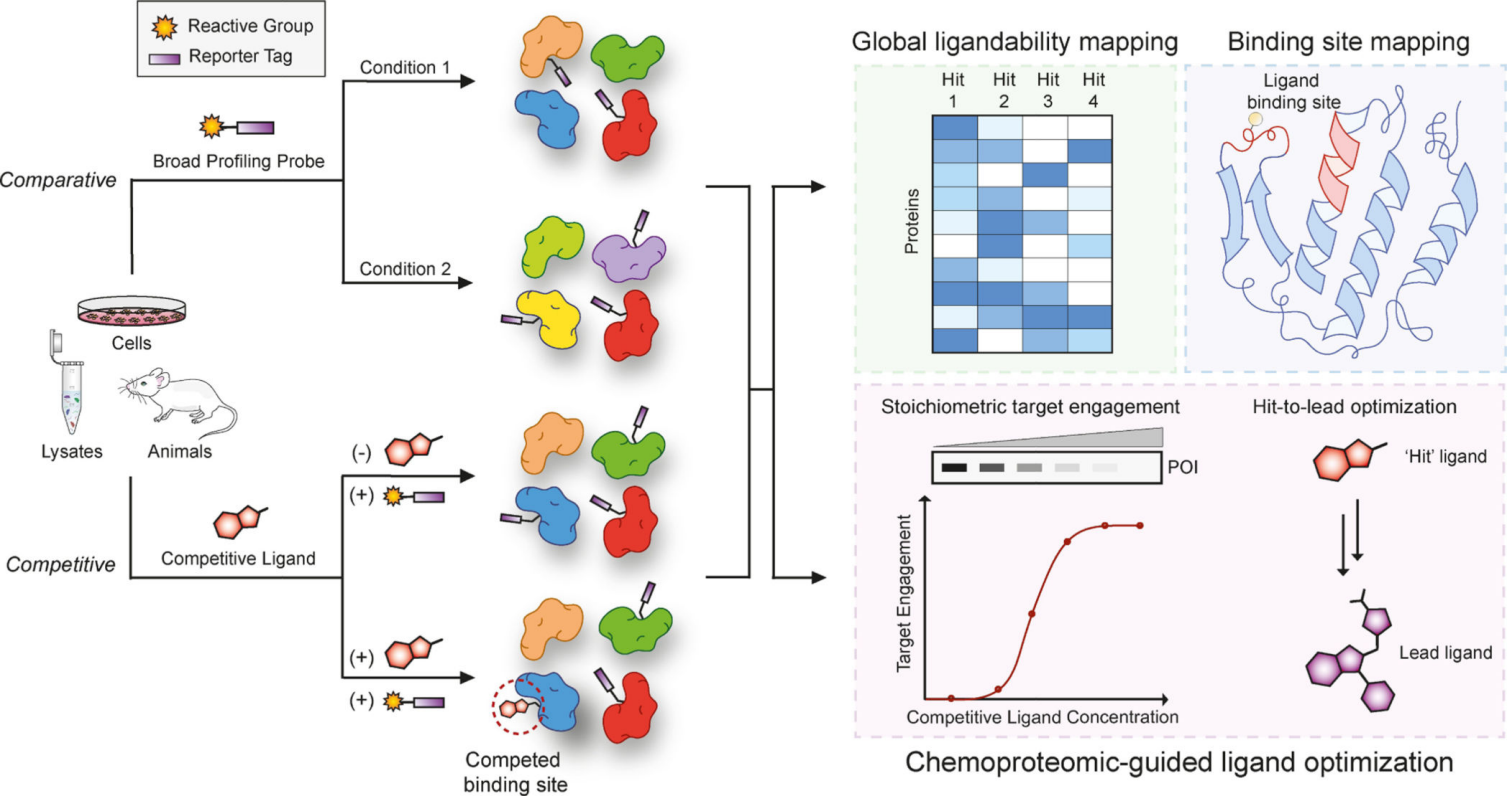
Chemoproteomic methods for proteome-wide fragment-based target and ligand discovery. Experiments can be performed in a comparative format, in which protein labeling using broad profiling probes is compared across multiple conditions, or in a competitive format, in which samples are treated with competing tag-free ligands that block probe labeling of proteins. These strategies enable global ligandability mapping, ligand binding site determination, and identification of hit binders that can further be optimized into selective leads that modulate protein function.

**Figure 2.**
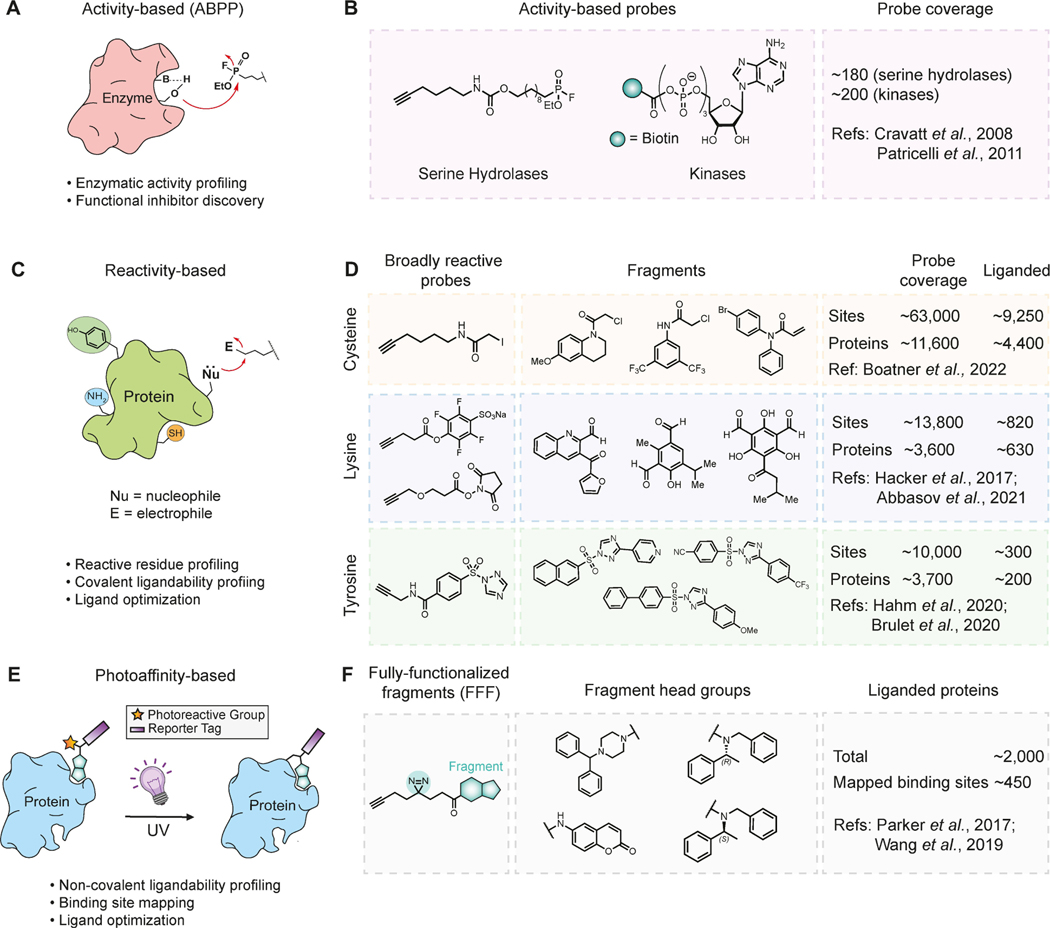
Classification of proteome-wide ligand discovery methods, probe examples and corresponding estimated coverages. (A) Activity-based protein profiling (ABPP) utilizes active-site directed probes (B) to report on enzymatic activity (e.g., serine hydrolases, kinases), allowing for their profiling in native systems and the development of inhibitors. (C-F) Examples of broad-profiling chemoproteomic platforms that have been utilized for proteome-wide fragment-based ligand and target discovery. (C-D) Various reactivity-based probes have been developed that target reactive residues (e.g., cysteine, lysine, and tyrosine) to uncover ligandable binding sites, independent of protein function. (E-F) Photoaffinity-based protein profiling allows for broad profiling of any residue through non-covalent interactions, bypassing the requirement of proximal reactive residues or protein activity. Fully-functionalized fragments (FFFs) are photoaffinity probes that enable ligandability profiling of non-covalent interactions.

Pioneered by the Cravatt Lab, activity-based protein profiling (ABPP) is the first strategy for the global mapping of ligandable proteins and corresponding identification of ligands directly in native systems (Figure 2A, B).^[11–12]^ Early ABPP applications employed electrophilic chemical probes that mechanistically react with the active sites of members of related enzyme families, serving as a reporter of their enzymatic activity.^[12]^ These probes enable functional profiling of many enzymes in parallel, facilitating the discovery of dysregulated enzymes in different biological states or systems via comparative profiling,^[13–16]^ and the development of inhibitors via competitive profiling of reactive chemical libraries (Figure 1).^[17]^ ABPP strategies have been developed for many enzyme classes, including serine hydrolases,^[11,18]^ cysteine proteases,^[19]^ metallohydrolases, phosphatases, deubiquitinating enzymes,^[20–21]^ kinases,^[22–23]^ and have been extensively reviewed elsewhere (Figure 2A, B).^[12,17,24–26]^

More recently, ABPP principles have been extended beyond enzyme active site profiling towards the global analysis of amino acid reactivity through the employment of electrophilic chemical probes that react with nucleophilic side chains (Figure 2C, D). These reactivity-based probes bypass the requirement of enzymatic activity for protein capture, thus dramatically expanding the scope of ABPP to all sites on proteins with reactive side chains. Among the twenty canonical amino acids, cysteine is the most intrinsically nucleophilic, offering a distinct reactive handle on proteins. Cysteines frequently play major roles in various enzymatic functions including catalysis, redox maintenance, and other complex biological processes, making them attractive targets for chemical probe development. In the first example of profiling the proteome-wide reactivity of proteinaceous nucleophiles, Weerapana and colleagues used tandem orthogonal proteolysis-ABPP (TOP-ABPP)^[27]^ for the simultaneous identification of protein targets as well as the exact sites of modification by a diverse set of electrophilic alkyne containing click-probes.^[28]^ The subsequent development of isotopic tandem orthogonal proteolysis – activity-based protein profiling (isoTOP-ABPP) enabled quantitative profiling of reactive cysteines using broad reactive iodoacetamide (IAA) – based probes^[29]^ (Figure 2D). Since the development of isoTOP-ABPP, numerous chemoproteomic investigations have led to the discovery of thousands of chemically reactive cysteine residues.^[29–33]^ Indeed, a recent analysis of published aggregated chemoproteomic data sets revealed that an impressive ~60,000 reactive cysteine residues across 11,000+ endogenous human proteins are chemically accessible using ABPP methods,^[34]^ highlighting the broad reach of this powerful technology to monitor cysteine status throughout the proteome. Broadly reactive chemoproteomic probes have been developed for other amino acids, including lysine,^[35–37]^ aspartate/glutamate,^[38–39]^ methionine,^[40]^ and tyrosine^[41–46]^ and more recent efforts have led to the identification of new reactive probes for nine unique amino acids as well as reactive *N*-termini for proteome-wide investigations (Figure 2C, D).^[47]^ Finally, the general principles of ABPP have also been extended for the broad surveyal of non-covalent, proteome wide interactions using photo-activatable probes (Figure 2E, F).^[48–53]^ In the sections below, we will discuss examples of how this powerful tool chest has been deployed to generate chemical probes for challenging protein targets, and more importantly, how it has expanded the boundaries of the ligandable proteome.

### Integration of Chemoproteomic Profiling with Fragment-Based Ligand Discovery

In contrast to traditional high-throughput screening approaches, which often consist of evaluating large chemical libraries against purified proteins, ABPP and related chemoproteomic methods enable the parallel screening of chemical libraries across hundreds to thousands of endogenous proteins directly in native biological systems. In doing so, chemoproteomic ligand and target discovery methods evaluate protein tractability to small molecules on a global scale, while also facilitating the development of ligands that can be fashioned into selective chemical probes. However, a major drawback of such approaches is the limited throughput nature of mass spectrometry (MS)-based workflows, restricting the number of library members that can be evaluated proteome-wide. Accordingly, the recent integration of fragment-based ligand discovery (FBLD) with chemical proteomic methods has enabled the global mapping of ligandable proteins with relatively small chemical libraries.^[48–49,54]^

Unlike chemical libraries used in most HTS, FBLD typically involves assaying smaller libraries (~1000) of low-molecular weight (<300 Da) compounds, i.e., fragments, for binding to purified protein targets.^[8–9,55–57]^ By setting low molecular weight limits, FBLD reduces the total possible number of atomic combinations by tens of orders of magnitude compared to traditional molecular weight cutoffs used for HTS.^[58]^ Fragment screens accordingly enable the exploration of a larger fraction of chemical space with a much smaller and more simplified library of compounds that tend to have superior ligand efficiencies compared to HTS hits.^[57]^ Fragment screens tend to have higher hit rates than HTS, but, due to the low-affinity of these hits, FBLD has been limited to the study of purified protein targets, where ligand-protein interactions are characterized by biophysical methods such as NMR and X-ray crystallography.^[59]^ However, by appending fragments to photo-activatable or electrophilic reactive groups, low-affinity fragment-protein interactions can be captured and processed using chemoproteomic workflows.^[49,54]^ In the following sections, we will discuss applications of this integrated approach for the global identification of both covalent and non-covalent ligand-protein interactions.

### Covalent Ligandability Mapping

Covalent ligands are attractive starting points for chemical probe development as they have the potential to achieve improved potency for shallow binding pockets and increased residency time compared to non-covalent counterparts.^[60]^ Driven, in part, by the emergence of robust chemoproteomic methods, like ABPP, to detect proteome-wide covalent interactions, there is increasing interest in the development of covalent compounds, both in academia and the pharmaceutical industry, underscored by the recent FDA approval of several covalent drugs, such as ibrutinib (Bruton’s tyrosine kinase inhibitor) and sotorasib (KRAS^G12C^ inhibitor).^[60]^ One of the first covalent-fragment screening approaches entailed incubating a library of disulfide-containing fragments with a purified protein containing either a native cysteine or recombinantly introduced cysteine within a binding site, resulting in disulfide exchange with the target cysteine and selection for fragments that are reversibly stabilized in its vicinity.^[61–62]^ Additional MS-based strategies have also been used to screen other electrophilic fragments against purified proteins.^[63–64]^

In pioneering studies from Backus *et al.*, covalent FBLD was integrated with quantitative proteomics to enable proteome-wide screening of cysteine-reactive fragments in cell lysates.^[54]^ Using a broadly reactive iodoacetamide alkyne probe to report on cysteine occupancy, a small library of ~60 diverse electrophilic fragments was screened proteome-wide using competitive isoTOP-ABPP, leading to the identification of over 750 ligandable cysteine across more than 620 proteins, including difficult-to-drug targets such as transcription factors and scaffold proteins. Here, the use of so-called ‘scout’ fragments enabled the broad surveyal of ligandable cysteine residues with a relatively small library. Critically, this study demonstrated that synthetic elaboration of such scout fragments into higher molecular weight compounds, guided by proteome-wide assessment of on and off-target reactivity, facilitated the development of more selective chemical probes. For example, chemoproteomic-guided ligand optimization efforts led to the development of selective covalent inhibitors of pro-CASP8 (Table 1, entry 1).^[54]^ In subsequent studies, Bar-Peled *et al.* investigated the cysteine reactivity differences in KEAP-1 mutant non-small cell lung cancer (NSCLC) cells with covalent scout fragments, which led to the mapping of 156 ligandable cysteines whose reactivity is regulated by NRF2.^[65]^ This discovery led to the development of a covalent ligand that targets one of these cysteines, C274 of the atypical orphan nuclear receptor NR0B1, resulting in disruption of a multimeric protein complex and subsequent suppression of anchorage-independent growth of KEAP1-mutant cancer cells (Table 1, entry 2). In similar studies, reactive scout fragments were deployed to broadly identify changes in cysteine ligandability as a function of human T-cell activation.^[30]^ Here, a total of ~16,000 reactive cysteines were identified and ~3,400 cysteines across ~2,200 proteins were discovered to be ligandable with scout fragments and spanned diverse immunological functions and protein classes. Notably, 160 proteins were found to harbor cysteines whose reactivity changed as a consequence of T cell activation, many of which were also ligandable with covalent fragments, highlighting the potential to develop covalent chemical probes for these targets under specific immune-relevant conditions. In addition to scout fragments serving as leads themselves for further optimization, they can also provide evidence of chemical tractability of specific binding sites to support targeted screens of larger, more elaborated compounds. Towards this end, in a recent study by Kavanagh *et al.*, C817 located in a non-catalytic pseudokinase domain of JAK1 was found to be ligandable with electrophilic scout fragments.^[66]^ The authors subsequently pursued a targeted MS-based screen with a larger library of structurally elaborate electrophiles, leading to the discovery of a highly selective compound targeting this allosteric site resulting in the blockade of JAK1-dependent cytokine signaling. These results underscore the utility of fragments to identify novel druggable binding sites on proteins, even if they do not serve as leads themselves for further optimization.

Though covalent fragment discovery has most frequently been pursued with cysteine-reactive libraries, this approach can also be applied to target other amino acid side chains. Lysine represents a particularly attractive target for covalent ligand development due to its abundance in proteins (~6% of all residues), particularly at enzyme active sites and PPIs as well as their frequent post translational modification. Analogous to broad-spectrum cysteine-reactive probes (i.e., IAA), recently Hacker *et al.* developed a selective lysine-reactive probe, enabling proteome-wide lysine profiling.^[36]^ Subsequent competitive isoTOP-ABPP experiments with a small library (~30 members) of electrophilic amine-reactive scout fragments uncovered approximately 100 ligandable sites across the human proteome (Figure 2D). It was also shown that these interactions could be advanced to covalent inhibitors of enzymes and protein-protein interactions. For instance, the authors demonstrated that binding of a fragment-like hit to K188 located in an allosteric binding site of PFKP effectively blocked its enzymatic activity (Table 1, entry 7). Additional efforts using an N-hydroxysuccinimide (NHS) ester-based click probe as well as NHS-based library members also enabled proteome-wide lysine profiling, though NHS ester-based probes tend to also have broader reactivity for serine, threonine, tyrosine, and cysteine.^[37]^ In recent work by Abbasov *et al.*, a larger library of electrophilic fragments (~180) across ~30 diverse aminophilic chemotypes were used to map ligandable lysines across human cancer cell lines and human immune cell proteomes.^[35]^ These efforts led to an impressive expansion of mapped reactive lysine residues, to ~14,000, amongst which more than 800 were found to be ligandable (Figure 2D). Notably, some of these interactions were found to occur at RNA-protein interfaces, including covalent liganding of K150 in IFIT5 by a sulfonamide fragment, which resulted in the disruption of RNA-protein interactions in cells (Table 1, entry 8).

Beyond cysteines and lysines, recent studies have utilized sulfur-fluoride exchange (SuFEx) chemistry to develop electrophiles targeting tyrosine residues.^[41,44,46]^ In addition, by modifying the reactive sulfur-fluoride group used in SuFEx, the Hsu lab developed a reactivity-based modality termed sulfur-triazole exchange or SuTEx, enabling the selective targeting of tyrosine residues.^[42,45]^ Proteome-wide profiling of SuTEx-based electrophilic fragments led to the identification of an additional 1,500 reactive tyrosine residues (Figure 2D), ~30% of which were found to react with electrophilic fragments. In this study, Brulet *et al.* showcased the ability of a SuTEx-based fragment to specifically target phosphotyrosine site Y8 on GSTP1 in cells, and subsequent blockage of its catalytic activity (Table 1, entry 9).^[43]^ In addition to electrophilic fragments targeting nucleophilic amino acids, recent studies have unveiled that proteinaceous electrophiles can be targeted by nucleophilic fragments. For example, Fu *et al.* recently screened a library of nucleophilic fragments that selectively react with sulfenic acids in cells and identified 500 + ligandable oxidized cysteine residues across ~400 proteins. They further demonstrated that liganding of sulfenated C108 by a nitroacetamide fragment disrupts the interaction between HDGF oncoprotein and its receptor, affecting downstream phosphorylation (Table 1, entry 10).^[67]^ In a similar fashion, Wang *et al.* profiled a small library of nucleophilic fragments (e.g., hydrazines, hydroxylamines) functionalized with alkyne handles, revealing differential reactivity across 100+ proteins in cells, targeting electrophilic modifications (e.g., glyoxylyl) and cofactors (e.g., pyruvoyl).^[68]^

### Non-Covalent Ligandability Mapping

Not all ligandable sites on proteins possess nucleophilic residues that can be targeted by electrophilic reactive groups, prompting the need for alternative chemoproteomic strategies to broadly assess non-covalent ligandability. Classical approaches to identify non-covalent small molecule-protein interactions, such as affinity chromatography, have been used to identify target proteins of bioactive compounds for decades.^[69]^ While affinity purification is well suited for identifying abundant and high-affinity targets, such methods are ill-suited for low affinity interactions (e.g., fragments) and low abundance protein targets. Photoaffinity labeling provides a practical solution as it allows transient reversible interactions between a probe and protein to be covalently captured upon UV-irradiation (Figure 2E). Though applications of photoaffinity probes date to the early 1960’s,^[70]^ most focus on the retrofitting of bioactive compounds, such as drugs, natural products, metabolites, or phenotypic screening hits, with photo-affinity labels to identify potential protein targets responsible for their observed biology.^[71–79]^

In order to broadly identify ligandable proteins, independent of the presence of reactive residues, specialized small molecule fragments equipped with photoaffinity enrichment handles have been integrated with ABPP methods for the global mapping of non-covalent ligand-protein interactions.^[49]^ In the first example of this approach, a library of 12 fully-functionalized fragment (FFF) probes, which are small molecule fragments appended to a retrieval tag composed of an alkyne and photoactivatable diazirine, led to the identification of more than 2,000 ligandable proteins directly in live cells. FFF probes were shown to display proteome-wide binding preferences and it was demonstrated that they could be used to map binding sites on ~450 proteins directly in live cells through the identification of probe-modified tryptic peptides (Figure 2F). It was further shown that these broad fragment interaction profiles could be advanced to selective non-covalent ligands that modulate protein function. For example, lead scout fragments identified in comparative profiling studies were used as binding site reporter probes in competitive profiling experiments in live cells (Figure 1) to develop inhibitors for the metabolic enzyme PTGR2 (Table 1, entry 3) and the mitochondrial acyl-carnitine transporter SLC25A20 (Table 1, entry 4). More recently, we applied this approach to develop first-in-class functional inhibitors of SLC15A4, an endolysosomal 12-transmembrane transporter essential for TLR7–9 and NOD signaling.^[80]^ Given that the molecular details of how SLC15A4 regulates TLR signaling are unclear, an integrated approach to identify SLC15A4 FFF binders that can also disrupt endolysosomal signaling directly in primary human immune cells was developed. After chemoproteomic-guided optimization, it was shown that the lead compound (Table 1, entry 5) inhibited SL-C15A4-mediated inflammatory cytokine production in a variety of immune model systems, including in lupus patient immune cells and *in vivo.* This study highlights the value of such platforms to identify small molecule modulators of challenging protein targets directly in translatable model systems and in a mechanistically agnostic fashion. To accelerate the screening of photoactivatable fragment libraries, this FFF approach has recently been adapted to target-based binding assays using recombinant purified proteins to identify ligands for targets such as KRASG12D and BCL6.^[81–82]^

Though FFFs yielded an abundance of potentially ligandable protein targets, due to the simple nature and promiscuity of fragments, discerning specific, authentic interactions from less specific or non-specific interactions required careful manual review. To overcome this challenge, a second-generation library of FFF probes was constructed, using a set of enantiomerically matched photo-crosslinking chiral fragment probes (‘enantioprobes’, Figure 2F).^[48]^ Here, it was rationalized that enantioprobes would possess similar physicochemical properties and share general non-specific proteomic interactions, therefore any identified stereoselective ligandable interaction would provide instant evidence of a specific interaction between the fragment and proteins in cells. Using a library of eight enantiomerically matched FFF probes, ~180 proteins displayed stereoselective binding preferences in cells, which included not only enzymes, but also adaptor proteins, transcription factors, scaffolding proteins and other tough-to-drug protein targets, as well as binding pockets with established small molecule ligands. This study highlights the integration of broad-profiling chemoproteomic methods with stereochemically enriched libraries^[83]^ as a powerful strategy to not only augment the discovery of selective probes, but also to expedite the global mapping of chemically tractable binding sites in native systems.

Although photoaffinity-based strategies, like FFFs allow for, in theory, the exploration of any small-molecule binding pocket, independent of protein function or neighboring residues, in live cells, some technical challenges remain that encumber their broad utilization for ligandability mapping. First, unlike probes that react selectively with specific amino acid side chains, allowing for direct insight into potential binding pockets, photoreactive groups, like diazirines, typically undergo insertion reactions with any neighboring X-H bond. Though recent studies have aimed to understand the reactivity of these groups,^[84–85]^ their application for binding site determination can be confounded by many factors, such as low abundances of probe-labeled peptides, co-fragmentation of probe adducts with peptide backbones,^[86]^ and the promiscuous nature of diazirine insertion into proteins.^[84–85,87]^ Second, the addition of photoaffinity and retrieval groups to fragments and other ligands can have substantial impact on their proteomic interactions,^[50]^ necessitating careful analysis of their proteomic profiles, particularly when utilizing photoaffinity ligands as leads for further development. Finally, unlike many reactivity-based probes that engage broad swaths of reactive cysteines or lysines, there is no universal probe that can serve as a reporter for the majority of small molecule binding sites throughout the proteome. Thus, fragment-based photoaffinity libraries should incorporate design principles to maximize their coverage of biologically relevant chemical space.^[88–90]^

### Phenotypic Screening of Chemoproteomic-Enabled, Fragment-Like Libraries

Though the broad profiling of scout fragments provides valuable insight into the potential tractability of ligandable proteins and binding sites, progressing simple scaffolds into more advanced chemical probes can be confounded by low-affinity and promiscuity of initial hits, potentially necessitating more labor-intensive optimization. Thus, in an alternative approach, chemoproteomic methods can be integrated with phenotypic screening to identify the targets of bioactive electrophiles. The coupling of phenotypic screens with chemoproteomic-enabled libraries affords the opportunity to not only discover compounds that modulate potential therapeutically translatable functional outcomes, but also the ability to directly identify the pharmacologically responsible target(s) without the need to derivatize hit compounds with affinity handles for target identification.^[79]^ Perhaps one of the first examples of this approach was the screening of a library of elaborated (median MW of molecular recognition group ~267 Da) fully-functionalized fragments (FFFs) to identify compounds that promoted adipogenesis.^[49]^ Chemoproteomic studies of a top hit (CPAG-1) led to the identification of relatively uncharacterized PGRMC2 as the primary target (Table 2, entry 1). It was subsequently found that CPAG-1 is a gain-of-function ligand and PGRMC2 is a molecular chaperone for heme, critical for adipocyte function.^[91]^ Recent studies have also demonstrated that structurally simple electrophilic compounds can be utilized in phenotypic screens and coupled to ABPP for immediate target identification. For example, RTN4 (Table 2, entry 2)^[92]^ was found to be the target of a structurally simple acrylamide that blocks colorectal cancer growth. Additionally, in a phenotypic screen of cysteine and lysine-reactive electrophiles to identify compounds that activate autophagy, a lead hit was found to target C277 in the catalytic subunit of the vATPase ATP6V1A, resulting in suppression of mTORC1 signaling and improved clearance of TDP-43 aggregates (Table 2, entry 3).^[93]^ More recently, in effort to identify compounds that modulate T cell function, a ~130 member library of fragment-like, as well as structurally elaborate cysteine reactive compounds, was screened for their suppressive effects on T cell activation, leading to the identification of several hits, including novel covalent inhibitors of TMEM173/ STING and the helicase ERCC3, amongst others (Table 2, entry 4).^[30]^

### Proteome-Wide Fragment Ligandability Mapping – Beyond Inhibitor Development

Proteome-wide fragment-based ligand discovery not only facilitates the identification of compounds that directly alter protein function (for example, blocking enzymatic activity or protein-protein interactions), but also the identification of ligands that bind protein targets without directly affecting their function. Such ‘silent’ ligands have the potential to be converted to compounds that might produce alternative outcomes on protein targets. Recently, fragment-based chemoproteomic strategies have been employed to identify silent ligands that can be co-opted into heterobifunctional molecules to alter protein homeostasis.

Targeted protein degradation (TPD) approaches utilize small molecules to induce proximity between components of the ubiquitin proteasome system (UPS), such as E3 ligases, and a target protein, resulting in its proteasome-mediated clearance.^[94–96]^ TPD strategies typically leverage two types of small molecules – molecular glues, which induce the formation of ternary complexes with E3 ubiquitin ligases and neosubstrates, and heterobifunctional molecules called proteolysis targeting chimeras (PROTACs), which link E3 ligase ligands to target-binding ligands, resulting in induced proximity.^[97]^ Though the application of PROTACs for TPD has emerged as a powerful strategy complementing conventional small molecule therapeutics and has been demonstrative as an effective approach to block functions of a diverse number of proteins,^[95]^ their development is bounded by the availability of ligands that can induce proximity between targets and the UPS system. Specifically, while there are *>*600 predicted E3 ligases in the human genome, only a handful have been found to be capable of supporting TPD and most studies leverage ligands targeting the E3 substrate recognition elements of Cereblon (CRBN) and von-Hippel Lindau (VHL).^[98–99]^

In this regard, chemoproteomic approaches integrating FBLD have recently been employed to expand the scope of E3 ligases.^[99–100]^ In one of the first examples of using chemoproteomics to discover E3 ligases that support TPD, electrophilic ‘scout’ fragments were appended to an FKBP12 ligand to identify covalent ligand-protein pairs that result in TPD. These efforts led to the discovery of a covalent fragment that binds the poorly characterized nuclear CUL4-DDB1 E3 ligase, DCAF16, that when coupled to selective ligands for intracellular proteins (for example, those that bind FKBP12 or BRD4), induces their proteasome-mediated degradation (Table 3, entry 1). It was further demonstrated that DCAF16-utilizing PROTACs selectively degrade nuclear target proteins, likely due to its nuclear localization, highlighting the potential of chemoproteomic ligand discovery methods to enable organelle-restricted TPD. In an extension of this approach, Zhang *et al.* developed a functional screening strategy to agnostically identify covalent ligands that support TPD.^[101]^ Here, the authors discovered an electrophilic fragment targeting DCAF11 which when incorporated into PROTACs, enabled the selective proteasomal degradation of targeted proteins, such as FKBP12 and the androgen receptor (AR). This approach has also been applied on purified protein, leading to the discovery of covalent E3 recruiters for RNF4 (Table 3, entry 2),^[102]^ RNF114 (Table 3, entry 3),^[103]^ and FEM1B E3 ligases (Table 3, entry 4).^[104]^ It is also worth noting, estimates based on aggregated published ABPP data sets suggest that 650+ E3 ligases possess cysteine residues that are potentially ligandable, underscoring the value of such chemoproteomic ligand discovery methods in the field of TPD.^[100]^ Recently, Henning *et al.* applied a similar chemoproteomic strategy to identify covalent ligands for deubiquitinases (DUBs) to be incorporated into bifunctional molecules, called DUBTACs, capable of inducing protein stabilization.^[105]^ Here the authors demonstrated that an electrophilic fragment-like compound that engages noncatalytic C23 of OTUB1 can be directed to the cystic fibrosis transmembrane conductance regulator (CTFR), resulting in increased protein stability (Table 3, entry 5).

Together, these examples highlight the utility of broad-profiling chemoproteomic approaches when integrated with proximity-based chemical probes. In this context, we envision that such chemoproteomic strategies will not only facilitate the discovery of ligands for additional PTM enzymes to co-opt into proximity-based molecules, but also for the systematic discovery of small molecule binders of challenging protein targets to enable their selective modification.

### Summary and Outlook

**Figure 3.**
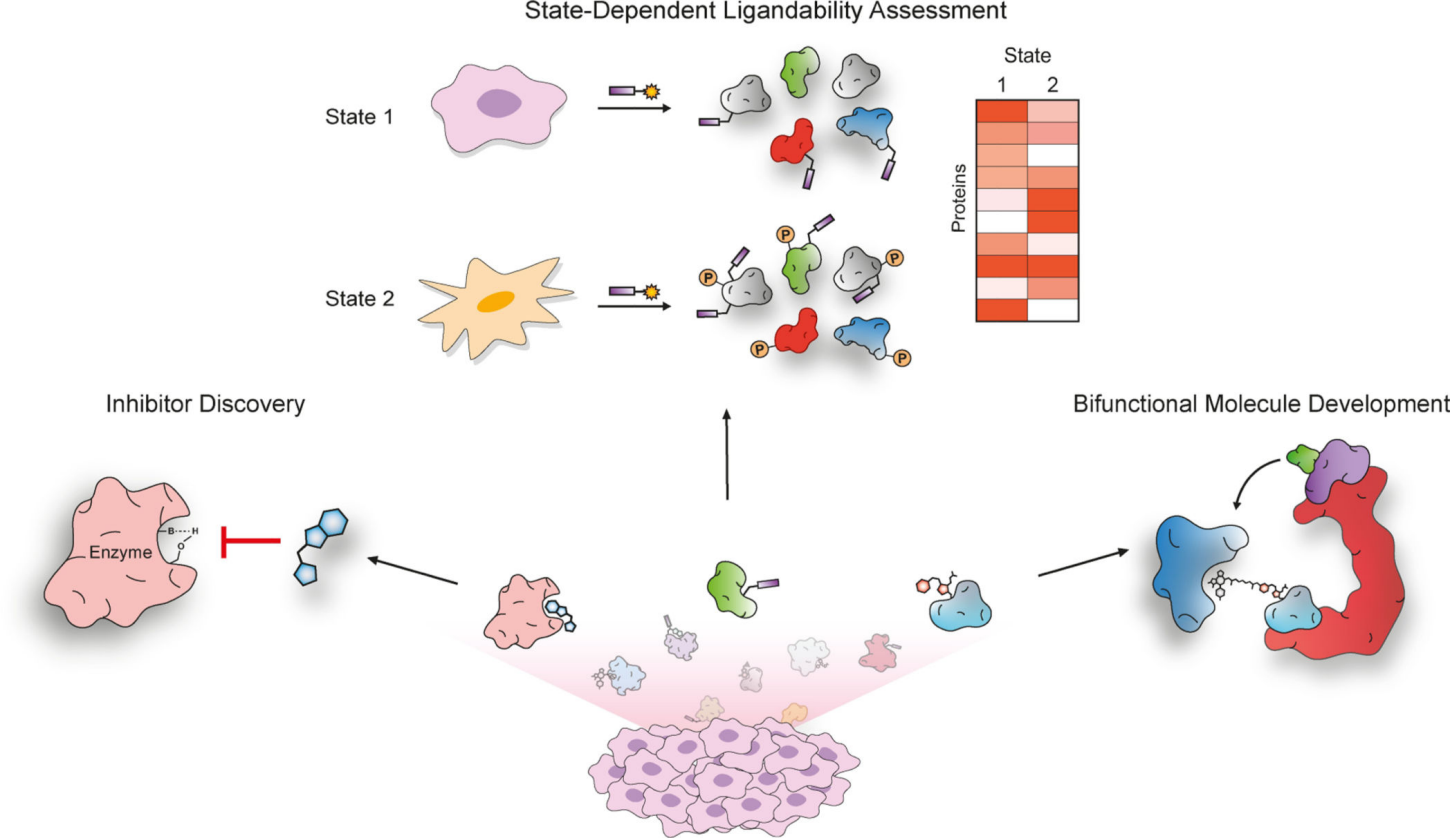
Applications of global ligandability studies. In addition to inhibitor discovery, fragment-based proteome-wide ligandability mapping also facilitates bifunctional molecule development (e.g., TPD), as well as the surveyal of state-dependent changes in proteome-wide ligandability.

In this review, we have discussed recent advances in the field of chemoproteomics that enable the discovery of ligands for proteins on a global scale. Specifically, the integration of principles borrowed from FBLD with powerful chemoproteomic methods like ABPP allow for the broad profiling of fragment-like libraries across thousands of proteins directly in native environments. These methods not only provide experimental evidence of proteins, and sites on proteins, that are capable of binding to drug-like small molecules, but they also yield a diverse set of ligands that can serve as chemical tool leads in a variety of applications (Figure 3). We’ve highlighted examples wherein proteome-wide fragment-based ligand discovery has been applied to develop first-in-class chemical probes for technically challenging targets and proteins that lack clear screenable functional readouts, as well as the recent employment of such methods to augment the development of heterobifunctional molecules for protein degraders or stabilizers. Additionally, the employment of ‘chemoproteomicenabled,’ fragment-like compounds permit the phenotypic screening of relatively smaller chemical libraries wherein hit compounds can be used directly to identify pharmacologically relevant targets. Finally, recent studies suggest that broad profiling ligandability platforms, such as proteome-wide fragment screening, can be used to unearth state-dependent ligandability events,^[30,65,106]^ providing a potential template for the discovery of chemical probes, and therefore therapies, that can selectively modulate protein function in a context dependent (e.g., diseased vs non-diseased) fashion.

Although this chemoproteomic screening strategy offers advantages for discovering compounds that target challenging protein targets in their native environments, progressing initial fragment leads into more potent and selective ligands is still a challenge. Often fragment hits have low affinities and are naturally promiscuous binders, confounding interpretation of their proteome-wide binding interactions and assessing the tractability of their binding sites. Towards this end, we highlighted the utility of stereochemically defined libraries to identify binding interactions that display preferences for one stereoisomer over others, thereby increasing confidence that the identified interaction is specific. Further, the limited throughput capacity of MS-based workflows restricts the number of compounds that can be screened against a given target once an initial fragment hit has been found. In this vein, the recent development of screening strategies that convert scout fragments into binding-site reporter probes (Table 1, entry 6)^[107]^ will substantially expedite optimization efforts by allowing the screening of larger chemical libraries to identify higher affinity compounds. We envision that continued advances and applications of this field will deliver useful chemical probes for investigations of human biology and therapeutic development, and ultimately furnish a complete portrait of the ligandable proteome.

## Figures and Tables

**Table 1.** Examples of fragment hits obtained through proteome-wide ligandability profiling.

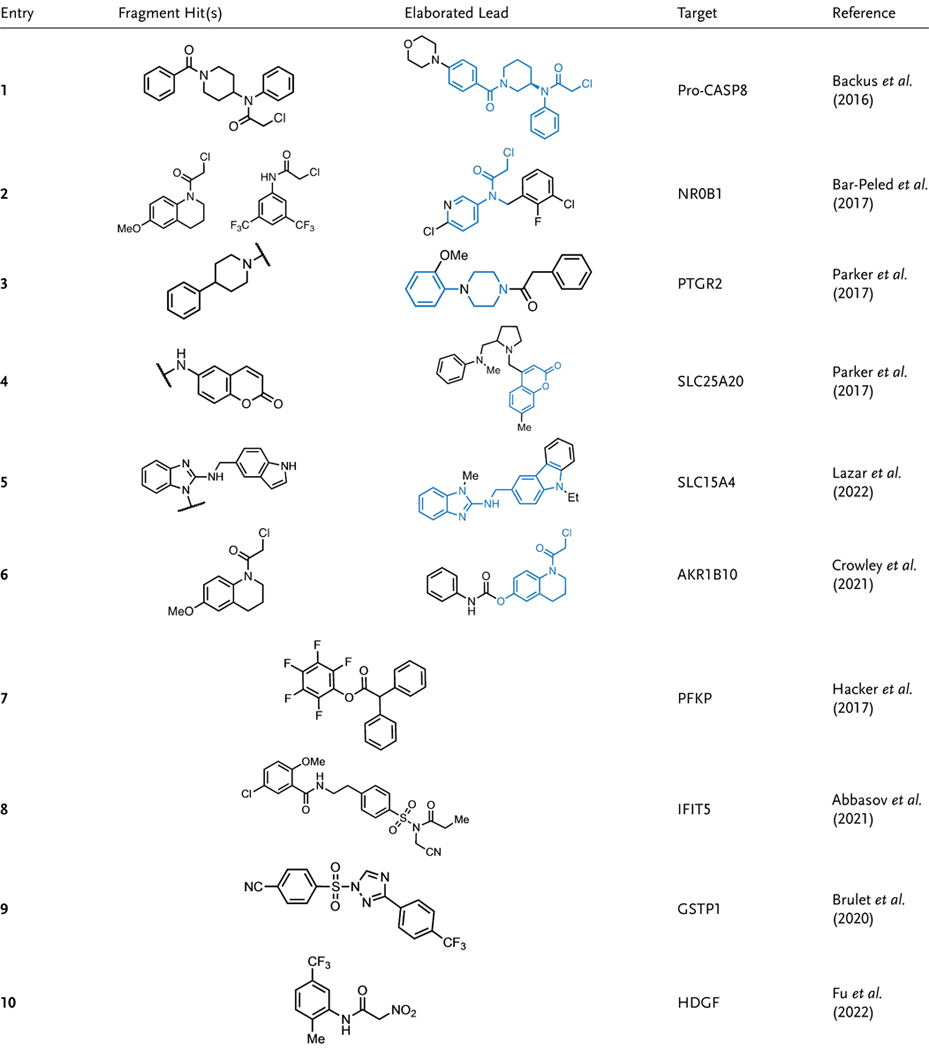

Entries 1–6: fragment hits and corresponding elaborated leads. Original fragment scaffolds are highlighted in blue. Entries 7–10: fragment-like hits used without further optimization.

**Table 2.** Examples of functional fragment-like hits obtained through phenotypic screening integrated with chemoproteomics.

Entry	Fragment Hit	Screen	Target	Reference

**1**	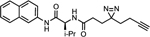	Adipocyte differentiation	PGRMC2	Parker *et al.* (2017)
**2**	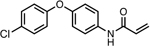	Cancer cell viability	RTN4	Bateman *et al.* (2017)
**3**	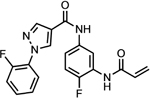	Autophagy induction	ATP6V1A	Chung *et al.* (2019)
**4**	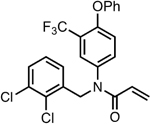	T cell activation/suppression	ERCC3	Vinogradova *et al.* (2020)

**Table 3.** Examples of fragment hits integrated into bifunctional molecules.

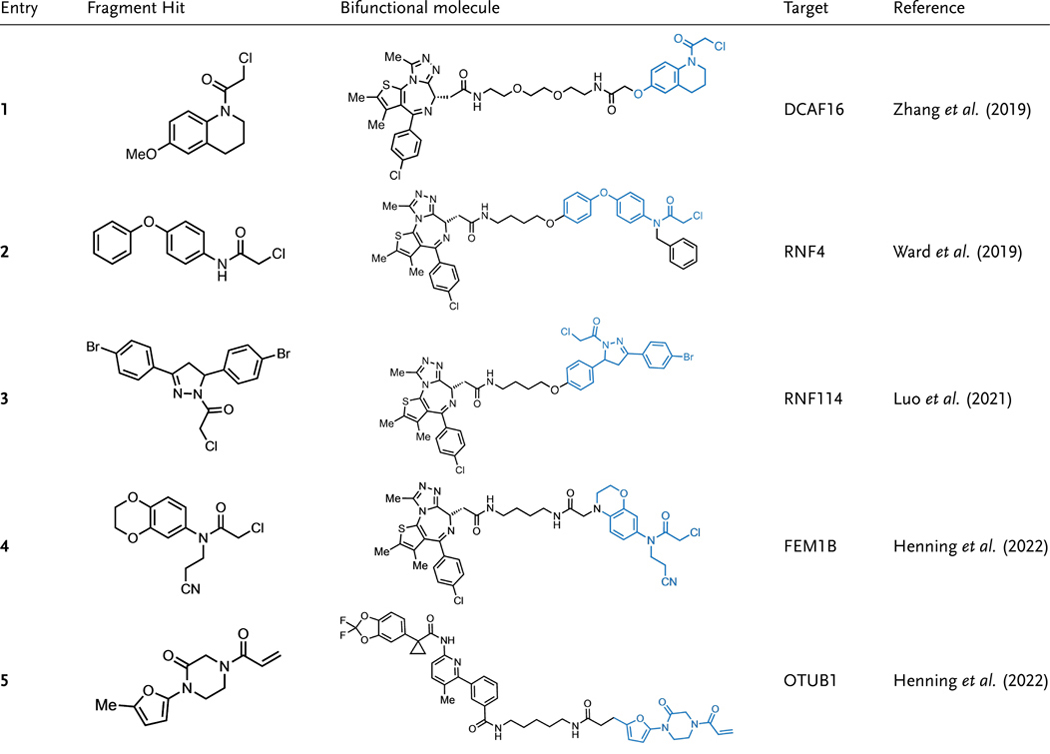

Original fragment scaffolds are highlighted in blue.

## Data Availability

Data sharing is not applicable to this article as no new data were created or analyzed in this study.
